# Supplementary material for: Hepatitis C Virus Infection Associated with Oral Potentially Malignant Disorder, Oral Cancer, and Liver Diseases: A Community-Based Cross-Sectional Study
Source: Cancers (Basel). 2025 Nov 18;17(22):3695. doi: 10.3390/cancers17223695 (PMC12650934; doi:10.3390/cancers17223695)
Supplement: Supplementary file 1 [file cancers-17-03695-s001.zip › cancers-3962225-supplementary.pdf]

Supplemental Table S1: The distribution of oral potential malignant disorder subtypes among participants.

**Classification**	**Number**	**%**
Leukoplakia	1287	70.9
Oral Submucous Fibrosis	285	15.7
Erythroleukoplakia	39	2.1
Verrucous Hyperplasia	39	2.1
Erythroplakia	32	1.8
Lichen Planus	10	0.6
Other Suspect OPMDs	124	6.8
Total	1,816	100.0%

Supplemental Table S2: Multiple multinomial logistic regression analysis of factors associated with subtypes of OPMDs, oral cancer, and cirrhosis or liver cancer.

Variables	OPMD												Oral Cancer			Cirrhosis or Liver Cancer		
	Leukoplakia			Epithelial Dysplasia, Erythroplakia, Erythroleukoplakia, Verrucous Hyperplasia			Submucous Fibrosis			Others								
	OR	95%CI		OR	95%CI		OR	95%CI		OR	95%CI		OR	95%CI		OR	95%CI	
Hepatitis C Viremia																		
No	Ref.			Ref.			Ref.			Ref.			Ref.			Ref.		
Yes	1.45	1.08	1.95	1.85	0.97	3.55	1.42	0.79	2.56	1.75	0.55	5.63	1.09	0.59	2.01	11.59	8.33	16.13
Age																		
30–44	Ref.			Ref.			Ref.			Ref.			Ref.			Ref.		
45–64	1.35	1.13	1.60	0.86	0.57	1.32	1.00	0.70	1.43	0.83	0.39	1.73	1.73	1.14	2.64	4.29	2.07	8.88
65+	0.80	0.63	1.02	0.48	0.26	0.88	0.72	0.45	1.16	0.54	0.20	1.46	1.69	1.03	2.78	1.73	0.75	4.00
Sex																		
Female	Ref.			Ref.			Ref.			Ref.			Ref.			Ref.		
Male	5.42	3.99	7.35	4.86	2.16	10.93	3.72	2.02	6.84	1.69	0.74	3.89	4.28	2.40	7.64	2.51	1.65	3.82
Education																		
College or above	Ref.			Ref.			Ref.			Ref.			Ref.			Ref.		
Senior High School	1.40	1.11	1.76	1.39	0.73	2.65	1.99	1.09	3.62	1.26	0.49	3.25	0.88	0.53	1.49	2.03	1.01	4.08
Junior High School	1.50	1.19	1.89	1.53	0.80	2.95	2.31	1.27	4.23	0.78	0.26	2.30	1.73	1.06	2.81	2.31	1.15	4.64
Elementary School or below	1.43	1.13	1.81	2.13	1.12	4.07	2.74	1.51	5.00	1.94	0.74	5.06	1.82	1.13	2.95	2.64	1.35	5.18
Smoking																		
Never	Ref.			Ref.			Ref.			Ref.			Ref.			Ref.		
Former	2.12	1.70	2.64	1.58	0.86	2.93	1.71	1.08	2.71	2.31	0.96	5.56	1.24	0.84	1.82	0.66	0.40	1.09
Current	5.05	4.21	6.07	4.25	2.57	7.02	2.96	1.98	4.41	3.85	1.85	8.02	1.57	1.11	2.23	1.02	0.70	1.50
Alcohol Consumption																		

Never	Ref.			Ref.			Ref.			Ref.			Ref.			Ref.		
Former	1.06	0.83	1.35	1.05	0.58	1.90	1.07	0.68	1.69	0.53	0.12	2.26	1.72	1.19	2.48	1.23	0.64	2.38
Current	1.14	1.00	1.31	1.04	0.74	1.46	1.17	0.89	1.54	1.46	0.80	2.67	1.76	1.37	2.28	1.31	0.90	1.91
Areca Nut chewing																		
Never	Ref.			Ref.			Ref.			Ref.			Ref.			Ref.		
Former	1.55	1.33	1.80	2.50	1.65	3.78	3.50	2.50	4.89	1.49	0.77	2.87	4.52	3.31	6.17	1.48	0.95	2.30
Current	2.24	1.92	2.63	4.23	2.83	6.34	4.91	3.48	6.95	1.02	0.44	2.37	4.12	2.90	5.85	1.85	1.17	2.95
BMI																		
Non-obesity (<30)	Ref.			Ref.			Ref.			Ref.			Ref.			Ref.		
Obesity (>=30)	1.68	1.40	2.03	0.95	0.54	1.65	1.06	0.69	1.63	1.89	0.86	4.18	1.22	0.82	1.83	1.26	0.72	2.18
Metabolic Syndrome																		
No	Ref.			Ref.			Ref.			Ref.			Ref.			Ref.		
Yes	1.37	1.21	1.55	1.41	1.03	1.93	1.32	1.03	1.70	1.18	0.68	2.06	1.09	0.86	1.39	0.77	0.55	1.08
